# Analysis of the direct economic impact of smoking-related hospitalizations in Italy

**DOI:** 10.18332/tid/188111

**Published:** 2024-06-03

**Authors:** Irene Possenti, Marco Scala, Magda Rognoni, Alessandra Lugo, Maria S. Cattaruzza, Sabrina Molinaro, Anna Odone, Luc J. M. Smits, Vincenzo Zagà, Silvano Gallus, Luca Cavalieri d’Oro

**Affiliations:** 1 Department of Medical Epidemiology, Istituto di Ricerche Farmacologiche Mario Negri IRCCS, Milan, Italy; 2 Epidemiology Unit, Local Health Authority (ATS - Agenzia per la Tutela della Salute) Brianza, Monza, Italy; 3 Department of Public Health and Infectious Diseases, Sapienza University, Rome, Italy; 4 Institute of Clinical Physiology, Italian National Research Council, Pisa, Italy; 5 Department of Public Health, Experimental and Forensic Medicine, University of Pavia, Pavia, Italy; 6 Department of Epidemiology, Faculty of Health, Medicine and Life Sciences, Maastricht University, Maastricht, The Netherlands; 7 Società Italiana di Tabaccologia, Rome, Italy

**Keywords:** Italy, tobacco, smoking, burden of disease, cost of illness

## Abstract

**INTRODUCTION:**

Tobacco-related diseases have a substantial economic impact in terms of medical expenses, loss of productivity, and premature death. Tobacco use is estimated to be responsible for more than 90000 deaths each year in Italy. We aimed to evaluate the annual direct economic impact on the National Health System of hospitalizations attributable to tobacco smoking in Italy.

**METHODS:**

We analyzed data from all the hospitalizations of patients aged ≥30 years that occurred in Italy for 12 selected tobacco-related diseases, during 2018. These diseases included oropharyngeal cancer, esophageal cancer, gastric cancer, lung cancer, pancreatic cancer, bladder cancer, laryngeal cancer, ischemic heart disease, stroke, diseases of arteries, arterioles, and capillaries, pneumonia and influenza, and chronic obstructive pulmonary disease. We obtained information on 984322 hospital discharge records, including each hospitalization's direct costs. Using relative risk estimates from the scientific literature, we computed the population attributable fraction for various tobacco-related diseases to estimate the economic impact attributable to tobacco smoking.

**RESULTS:**

One-third of all hospitalizations occurred in 2018 in Italy among people aged ≥30 years for 12 tobacco-related diseases were found to be attributable to smoking, accounting for a total cost of €1.64 billion. Among the diseases considered, those with the highest expenditures attributable to tobacco smoking were ischemic heart disease, cerebrovascular disease, and lung cancer, accounting for €556 million, €290 million, and €229 million, respectively.

**CONCLUSIONS:**

Tobacco has a substantial economic impact in Italy, accounting for around 6% of the total cost of hospitalizations in 2018. This figure is expected to be largely underestimated due to several conservative assumptions adopted in the statistical analyses. It is imperative to prioritize comprehensive tobacco control measures to counteract the huge healthcare costs due to tobacco smoking.

## INTRODUCTION

Tobacco smoke contains over 7000 chemicals, of which at least 250 are known to be harmful to health, and 69 are known carcinogens^1,2^. Despite widespread knowledge of the diseases caused by tobacco smoking, including cancer, ischemic heart disease (IHD), stroke, and chronic obstructive pulmonary disease (COPD), it remains the main avoidable risk factor, representing a serious public health problem with millions of people continuing to smoke globally^3^.

Tobacco smoking is the second leading cause of premature death and disability worldwide, with an estimated 8 million deaths per year attributable to its use, including more than 1 million deaths attributable to exposure to secondhand smoke (SHS)^3,4^. It is estimated that there were more than 100 million tobacco-related deaths worldwide in the 20th century, and if current trends continue, the projected number of tobacco-related deaths in the 21st century could reach 1 billion^5^.

The global economic expenditure attributable to tobacco smoking, which includes both direct health expenditure and indirect costs from productivity loss, is estimated at $1.4 trillion per year, or 1.8% of global gross domestic product (GDP)^4,6^. Global health expenditure attributable to tobacco-related diseases was calculated at $422 billion in 2012, representing 5.7% of global health expenditure^6^. The economic cost of tobacco smoking in the United States exceeded $600 billion in 2018, including more than $230 billion in direct healthcare expenditures and more than $370 billion in indirect expenditures due to productivity loss. Of these indirect costs, $185 billion were due to smoking-related illnesses, $180 billion to smoking-related premature deaths, and $7 billion to premature deaths from SHS exposure^1,7-9^.

In Italy, it is estimated that 96000 deaths per year are attributable to tobacco smoking, representing 15% of all deaths (20.6% of all deaths in men and 7.9% of all deaths in women)^10^. It has also been estimated that, without the implementation of effective tobacco control measures, nearly 300000 lives would be prematurely lost from smoking by 2040^11^. Smoking prevalence in Italy has been declining for a long time, but since 2013, the decrease has faltered, and there has been a worrying shift in the trend, with the prevalence of current smokers rising to 24.2% in 2022, the first increase since 2009^3,12^. The rise in smoking prevalence is partly due to the lack of adoption of effective tobacco control policies in Italy^13^ and to the introduction of novel nicotine-containing products, such as electronic cigarettes and heated tobacco products^14^. These novel products, which have become extremely popular in Italy, particularly among young people, have obstructed tobacco control, leading to a renormalization of the smoking habit^14,15^.

In Italy, healthcare services are provided by both public and private hospitals. Public hospitals are funded and managed by the government, offering public medical services to all citizens and residents, typically free or with minimal out-of-pocket expenses. The cost of treatment in public hospitals is covered by the National Health System, ensuring universal access to healthcare services. In contrast, private hospitals, alongside public treatments covered by the National Health System, may offer private higher cost treatment options, often with more amenities and comfort. Still, patients bear the total cost unless covered by private insurance.

This article examines the direct economic impact of tobacco-related hospitalizations on the National Health System in Italy. Previous information on smoking-related expenditure in Italy is limited. The most recent data on the issue are from 2009 and show that smoking-related diseases account for 6.3% of the total expenditure in the Italian National Health System^16^.

## METHODS

The data used in this study were provided by the Italian Ministry of Health. They were extracted from the Italian National Hospital Discharge Database (Hospital Discharge Records – ‘Schede di Dimissione Ospedaliera’, SDO), an electronic database with complete coverage of discharges from all public and private hospitals in Italy. Hospital discharge reports from the SDO were anonymized and included relevant information from the patient’s medical records, including personal data (i.e. sex, date, and place of birth, and region of residence), organizational arrangements of the stay (i.e. admission unit, months of hospitalization and discharge, length of stay, and cost of the hospitalization), and clinical information (e.g. primary diagnosis, five secondary diagnoses, and discharge modalities).

Hospitalization costs were expressed in euros and calculated according to the Diagnosis Related Groups (DRG) system used by the Italian National Health Service. Primary and secondary diagnoses were classified according to the International Classification of Diseases, Ninth Revision, Clinical Modification (ICD-9-CM)^17^.

The data provided by the Italian National Health Service covered all hospitalizations in 2018 for patients aged ≥30 years with one of 14 selected diseases as their primary diagnosis. We excluded two of the selected diseases (peptic ulcer and renal pelvic cancer), resulting in the following 12 tobacco-related diseases: oropharyngeal cancer (ICD-9-CM: 141, 143-146, 148, 149), esophageal cancer (150), gastric cancer (151), lung cancer (162), pancreatic cancer (157), bladder cancer (188), laryngeal cancer (161), IHD (410-414), stroke (430-438), diseases of arteries, arterioles, and capillaries (440-448, hereafter referred to as arteriosclerosis), pneumonia and influenza (480-487), and COPD (490-492, 496). In addition, hospital discharge reports with any tobacco-related cancer as a secondary diagnosis and a cancer-related treatment as the main diagnosis (e.g. chemotherapy or radiotherapy) were also included in the analyses. This exception was based on the recognition that the costs associated with these treatments were directly attributable to the underlying tobacco-related cancer, registered as a secondary diagnosis.

The number of hospitalizations and total costs were obtained for each of the 12 tobacco-related diseases considered in this study. To compute each disease-specific population attributable fraction (PAF), the following formula was applied to each sex and age subgroup^18^:


$$PAF=\frac{\left[(p0+p1\times RR1+p2\times RR2)\text{-}1\right]}{\left(p0+p1\times RR1+p2\times RR2\right)}$$

Where p0, p1, and p2 represent the prevalence of never smokers, current smokers, and former smokers, respectively, and RR1 and RR2 are the disease-specific relative risks (RR) for current and former smokers, respectively, compared with never smokers.

Data on the prevalence of never, former, and current smokers in each sex and age subgroup were obtained from the 2003 survey conducted by the Italian National Institute of Statistics (ISTAT)^19^ and are shown in Supplementary file Table 1. We assumed a 15-year lag between smoking and the onset of tobacco-related disease because of the expected long latency of several diseases, including cancer^20^. Disease-specific RRs for current and former smokers compared with non-smokers were derived from previously conducted meta-analyses and are detailed in Supplementary file Table 2. Additionally, sensitivity analyses were performed using different RRs derived from a pooled analysis^21^.

The number of hospitalizations and costs attributable to tobacco smoking was therefore calculated using the following formulas:


$$\text{Attributable hospitalizations}=\underset{i}{\Sigma }\underset{j}{\Sigma }\underset{k}{\Sigma }PAF_{ijk}\times numberofhospitalizations_{ijk}$$

$$\text{Attributable costs}=\underset{i}{\Sigma }\underset{j}{\Sigma }\underset{k}{\Sigma }PAF_{ijk}\times totalcosts_{ijk}$$

where i stands for sex, j for age group, and k for specific disease.

Analyses were performed using STATA, version 17 (StataCorp, College Station, Texas 77845, USA).

## RESULTS

A total of 984322 hospital discharge reports were included in the analysis, representing all the hospitalizations of patients aged ≥30 years, with one of the 12 considered tobacco-related diseases in Italy in 2018. These hospitalizations had a cumulative cost of €4.94 billion. Supplementary file Figure 1 shows the hospitalization rates per million inhabitants by the main diagnosis. Among these tobacco-related diseases, the most frequent hospitalizations were for IHD, stroke, pneumonia, and influenza, accounting for 263214, 251090, and 135782 hospitalizations, respectively (Table 1). Among tobacco-related cancers, bladder cancer accounted for the highest number of hospitalizations (77961), followed by lung cancer (68284) and pancreatic cancer (24507).

**Table 1:** Hospitalizations and expenditures, total and attributable to smoking, stratified by disease, Italy, 2018

*Disease*	*Total number of hospitalizations*	*PAF Hospitalization %*	*Attributable hospitalizations*	*Total expenditure €*	*PAF expenditure %*	*Attributable expenditure €*
Oropharyngeal cancer	9742	43.72	4259	46308439	43.87	20313925
Esophageal cancer	4505	41.76	1881	22684856	41.91	9507821
Gastric cancer	19869	17.69	3514	118904756	17.63	20962538
Pancreatic cancer	24507	19.52	4783	123040960	19.67	24199247
Laryngeal cancer	7754	74.77	5798	42172339	74.70	31504228
Lung cancer	68284	73.67	50308	310464131	73.76	228996823
Bladder cancer	77961	41.55	32396	252928438	41.69	105201584
IHD	263214	35.29	92900	1544608137	36.02	556423694
Stroke	251090	20.70	51986	1347555704	21.53	290079568
Arteriosclerosis	86132	37.76	32523	560205936	38.67	216617474
Pneumonia and influenza	135782	14.80	20102	481093909	15.01	72201303
COPD	35482	74.70	26504	87645877	75.41	66096567
Total	984322	3327	326954	4937613482	33.31	1642104772

Attributable expenditure: expenditure (€) attributable to tobacco smoking. Attributable hospitalizations: number of hospitalizations attributable to tobacco smoking. COPD: chronic obstructive pulmonary disease. IHD: ischemic heart disease. PAF: population attributable fraction.

**Figure 1:**
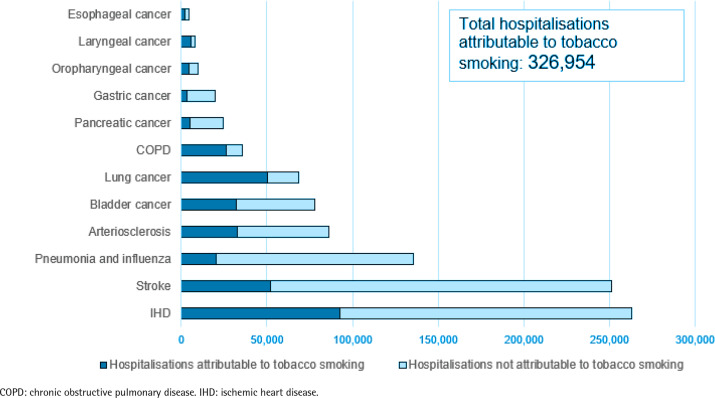
Disease-specific number of hospitalizations attributable and not attributable to tobacco smoking, Italy, 2018

Out of 984322 hospitalizations, 326954 were found to be directly attributable to tobacco smoking, accounting for 33% of the total number of hospitalizations considered (Table 1). Among the tobacco-related diseases considered, the highest PAF values, which represent the proportions of hospitalizations for the disease attributable to tobacco smoking, were observed for laryngeal cancer, with smoking contributing to approximately 75% of the reported hospitalizations, followed by COPD (75%) and lung cancer (74%). Among all hospitalizations for the considered tobacco-related cancers, 48% were found to be attributable to tobacco smoking. The highest number of hospitalizations attributable to tobacco smoking was observed for IHD, with 92900 hospitalizations, followed by stroke, with 51986 hospitalizations, and lung cancer, with 50308 hospitalizations (Figure 1). Tobacco smoking was responsible for 40% and 22% of the considered hospitalizations in men and women, respectively, while in both sexes, the age group most affected by smoking was those aged 60–69 years, in which 42% of the analyzed hospitalizations (86955 out of 209482) were attributable to tobacco smoking (Table 2).

**Table 2:** Hospitalizations, total and attributable to smoking, stratified by sex and age group, Italy, 2018

*Age (years)*	*Males*			*Females*			*Total*		
	*Tot H*	*Attr H*	*PAF %*	*Tot H*	*Attr H*	*PAF %*	*Tot H*	*Attr H*	*PAF %*
30–34	2352	380	16.13	1785	236	13.25	4137	616	14.89
35–39	4110	1352	32.90	2927	650	22.18	7037	2002	28.45
40–49	26426	10236	38.73	13675	3694	27.01	40101	13930	34.74
50–59	77598	32458	41.83	32749	10954	33.45	110347	43412	39.34
60–69	148437	66122	44.55	61045	20833	34.13	209482	86955	41.52
70–79	201098	83645	41.59	102975	26809	26.03	304073	110454	36.32
≥80	155776	52376	33.62	153368	17207	11.22	309144	69583	22.51
Total	615797	246569	40.04	368524	80383	21.81	984321	326952	33.22

Attr H: number of hospitalizations attributable to tobacco smoking. PAF: population attributable fraction. Tot H: total number of hospitalizations.

**Figure 2:**
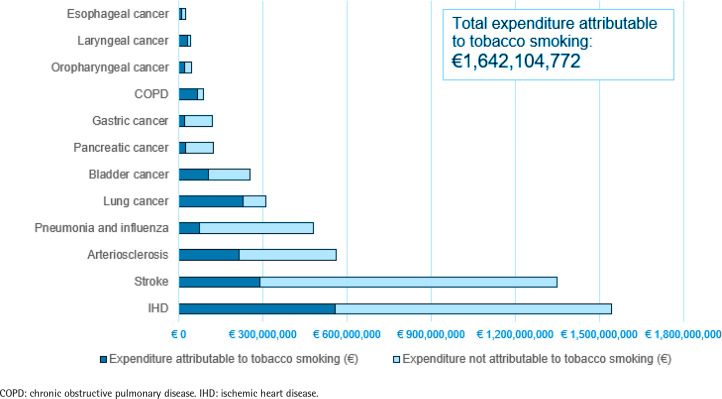
Disease-specific expenditure attributable and not attributable to tobacco smoking, Italy, 2018

The cumulative expenditure attributable to tobacco smoking reached €1.64 billion, accounting for approximately 33% of the total healthcare expenditure associated with the hospitalizations analyzed. The highest healthcare expenditure attributable to tobacco smoking was observed for IHD, with costs of €556 million, followed by stroke with €290 million, and lung cancer with €229 million (Figure 2 and Table 1). Tobacco smoking was responsible for 40% and 22% of the expenditure in men and women, respectively, while the age group most affected by smoking was those aged 60–69 years, in which 41% of the expenditure (457539 out of 1123058 thousand €) were attributable to tobacco smoking (Table 3).

**Table 3:** Expenditure (in thousands of €), total and attributable to smoking, stratified by sex and age group, Italy, 2018

*Age (years)*	*Males*			*Females*			*Total*		
	*Tot Exp*	*Attr Exp*	*PAF %*	*Tot Exp*	*Attr Exp*	*PAF %*	*Tot Exp*	*Attr Exp*	*PAF %*
30–34	11136	1882	16.90	7855	1051	13.38	18991	2932	15.44
35–39	21132	7214	34.14	12930	2909	22.49	34062	10693	29.87
40–49	147493	57283	38.84	69169	18252	26.39	216662	75535	34.86
50–59	438397	180732	41.23	165966	53490	32.23	604363	234222	38.76
60–69	813345	355596	43.72	309713	101943	32.92	1123058	457539	40.74
70–79	1058798	431524	40.85	521216	130817	25.10	1580014	562341	35.59
≥80	704347	231284	32.84	656116	68127	10.38	1360463	299411	22.01
Total	3194648	1276515	39.61	1742965	376589	21.61	4937613	1642673	33.27

Attr Exp: expenditure attributable to tobacco smoking. PAF: population attributable fraction. Tot Exp: total expenditure.

In the sensitivity analyses performed using different RRs, the number of hospitalizations attributable to smoking increased to 373621, representing 38% of the total number of hospitalizations considered. Similarly, the cumulative expenditure attributable to smoking increased to €1.84 billion, representing 37% of the total healthcare expenditure associated with the analyzed hospitalizations (Supplementary file Table 3).

## DISCUSSION

This study provides valuable insights into analyzing the burden of tobacco-related hospitalizations and associated healthcare costs in Italy. By analyzing a comprehensive dataset of almost 1 million hospital discharge reports for 12 selected tobacco-related diseases in 2018, we found that tobacco smoking was responsible for a third of the hospitalizations and expenditures associated with these diseases, amounting to more than 320000 hospitalizations and more than €1.64 billion.

The diseases with the highest population attributable fraction (PAF) in our study were chronic obstructive pulmonary disease (COPD), laryngeal cancer, and lung cancer, with smoking contributing approximately three out of four hospitalizations for each of these diseases. Our findings are partly aligned with those of two similar studies conducted in the UK and Switzerland, which found that lung cancer and COPD were among the diseases with the highest proportion attributable to smoking^22,23^. However, direct comparisons between studies should be made with caution because of differences in country-specific smoking prevalence and methodologies applied, including the reporting of combined results for several tobacco-related cancers and not for laryngeal cancer separately.

Our study revealed a substantial burden of tobacco-related hospitalizations in Italy. In particular, if tobacco smoking was eliminated, more than 300000 hospitalizations could be prevented each year. Among the 12 tobacco-related diseases considered, the highest numbers of hospitalizations attributable to tobacco were observed for IHD, stroke, and lung cancer. These findings reflect the significant impact of smoking on cardiovascular diseases and respiratory conditions.

In addition, our study revealed important sex differences, with a higher proportion of hospitalizations and expenditures attributable to tobacco smoking observed in men than in women. Overall, the number of hospital admissions and expenditures due to smoking is three times higher in men than in women. This is in line with previous studies conducted in European countries, although they focused on mortality rather than hospitalization^22,23^. Possible factors contributing to this difference include higher smoking prevalence rates and intensity of the smoking habit in men than in women.

The results highlight that tobacco smoking has a substantial economic impact in Italy, accounting for one-third of the direct expenditure for hospitalizations for the tobacco-related diseases considered. This finding is consistent with a 2017 study conducted in Switzerland, which reported that tobacco smoking accounted for 28% of the expenditure related to tobacco-related diseases^22^. Examining the total cost of hospitalizations in Italy in 2018^24^, our findings indicate that tobacco smoking accounts for at least 5.9% of the total expenditure.

The estimated expenditure to hospitalization attributable to tobacco smoking in our study is substantially underestimated. First, our analysis focused on only 12 specific tobacco-related diseases, opting for a conservative selection. However, recent literature has identified a total of 56 smoking-related diseases^25^. Notably, our selection of diseases, although conservative, still accounts for a substantial proportion, approximately 85%, of the global deaths attributed to tobacco smoking as calculated by the Global Burden of Disease Study^26^, suggesting that we have captured a substantial, albeit not wholly exhaustive, proportion of the total hospitalizations attributable to smoking. Despite this, our analysis did not encompass several common and costly diseases that have been demonstrated to be linked to smoking, such as breast and colorectal cancer^21,27,28^, which stand as the two most frequently diagnosed cancers in Italy^29^. We also did not include diabetes, prostate cancer, and liver cancer, all established to be linked with smoking^21^. Therefore, the economic impact of smoking on the healthcare system is likely to be higher when considering the broader range of tobacco-related diseases. Second, our analysis was mainly limited to hospitalizations for smoking-related diseases as the primary diagnosis. This approach did not consider hospitalizations where a tobacco-related disease was listed as a secondary diagnosis. As smoking may contribute to the severity and complications of several health conditions, this approach may have underestimated the actual economic expenditure attributable to tobacco smoking. Third, we calculated hospitalizations and costs directly attributable to active smoking and did not include an assessment of the impact of exposure to SHS, which would provide a complete understanding of the total burden of tobacco-related diseases in non-smokers. Notably, recent meta-analyses have shown significant associations between SHS exposure and the risk of cervical and breast cancer^30,31^. However, the health burden from SHS is expected to be relatively low, as it is estimated to account for less than 10% of the disability-adjusted life years attributable to active smoking in Italy^3^. Future studies should focus on estimating the direct economic costs attributable to SHS exposure in the non-smoking population.

It should also be noted that the costs estimated in our study represent only a fraction of the healthcare costs attributable to tobacco smoking. In fact, hospitalizations account for less than a quarter of the total costs incurred by the Italian National Health System, and we expect tobacco smoking to have a substantial impact on the costs of outpatient services, drugs, and other healthcare resources^28^. Moreover, our analysis focuses on the direct economic impact of tobacco-related hospitalizations on the National Health System. It does not take into account out-of-pocket expenditures incurred by individuals. This omission is particularly relevant for private hospitalizations, where patients bear the total cost of treatment. Future research should address this limitation by incorporating out-of-pocket costs to provide a more comprehensive understanding of the economic impact of tobacco-related morbidity. The non-inclusion of private hospitalizations, which do not affect the National Health System, may also have led to an underestimation of the number of hospitalizations attributable to smoking. However, it is essential to note that private hospitalizations represent only a tiny proportion of total hospitalizations in Italy. In addition, we did not account for the burden of indirect costs. Indirect costs, including productivity losses due to premature mortality or disability, informal care for people with tobacco-related illnesses, and the costs of tobacco-related fires and sickness absence, are essential components in assessing the overall economic impact of smoking on society. A recent study revealed that smoking costs England £49.2 billion each year in lost productivity and service costs, along with an additional £25.9 billion lost in quality-adjusted life years due to premature death from smoking^32^. These findings underscore the importance of incorporating such indirect costs in future studies to provide a more comprehensive assessment of the economic burden of tobacco smoking in Italy.

### Limitations

Several limitations should be considered when interpreting this study’s results. First, several assumptions were made in the calculation of the PAFs. Notably, the appropriateness of the relative risks (RR) employed warrants consideration, as specific RR estimates for Italy would be ideal. However, the evidence is derived from comprehensive meta-analyses and systematic reviews encompassing diverse populations, including Europe. It is also worth noting that results from sensitivity analyses suggest that the choice of RR may have led to an underestimation of the number of hospitalizations and attributable costs. Moreover, unmeasured factors, such as socioeconomic status and lifestyle habits, may influence both smoking behavior and hospitalization rates, potentially biasing our results. Finally, it should be noted that the results of our study are specific to the Italian context and may not be directly generalizable to other countries. Differences in healthcare systems, smoking prevalence, healthcare practices, and socioeconomic factors between countries may limit the applicability of our results to other countries.

## CONCLUSIONS

Given the substantial burden of tobacco-related hospitalizations and associated costs, it is essential to prioritize comprehensive tobacco control measures to reduce smoking prevalence. One particularly effective strategy is to increase tobacco excise taxes. Indeed, it is well established that in high-income countries, increases in taxation – and prices – reduce consumption while increasing revenues^33,34^. Particularly, it has been estimated that a 10% increase in prices would result in a 4% decrease in consumption^34,35^. Furthermore, given the fact that these costs are borne by the regions, whereas taxes on cigarette sales are collected by the Italian State, it might be worth exploring the possibility of giving the regions more control over tobacco taxation.

## Supplementary Material



## Data Availability

The data supporting this research cannot be made available for privacy or other reasons.
