# Supplementary material for: Analysis of the direct economic impact of smoking-related hospitalizations in Italy
Source: Tob Induc Dis. 2024 Jun 3;22:10.18332/tid/188111. doi: 10.18332/tid/188111 (PMC11145629; doi:10.18332/tid/188111)
Supplement: Supplementary file 1 [file TID-22-96-s1.pdf]

Supplementary Table 1 – Prevalence of current, former, and never smokers in the Italian population, by sex and age groups (ISTAT, 2003) [1]

Age group in 2003	Age group in 2018	% of current smokers	% of former smokers	% of never smokers
**Males**				
**15-19**	**30-34**	14.7	4.2	81.1
**20-24**	**35-39**	38.1	9.3	52.6
**25-34**	**40-49**	40.2	13.7	46.0
**35-44**	**50-59**	37.9	23.1	39.0
**45-54**	**60-69**	37.1	34.0	28.9
**55-64**	**70-79**	30.1	40.8	29.1
**65+**	**80+**	18.0	54.1	27.9
**Females**				
**15-19**	**30-34**	11.9	4.6	83.5
**20-24**	**35-39**	21.8	7.9	70.3
**25-34**	**40-49**	22.5	13.5	64.0
**35-44**	**50-59**	25.5	18.3	56.2
**45-54**	**60-69**	24.8	19.1	56.0
**55-64**	**70-79**	17.1	17.0	65.9
**65+**	**80+**	4.9	12.5	82.6

Supplementary Table 2 – Relative risks (RR) and 95% confidence intervals (CI) for current and former smokers compared to never smokers for each tobacco-related disease

Disease (ICD-9-CM codes) [source meta-analysis]	RR (95% CI)	
	Former smokers	Current smokers
**Oropharyngeal cancer (141, 143-146, 148-149) [2]**	1.40 (0.99-2.00)	3.43 (2.37-4.94)
**Esophageal cancer (150) [2]**	2.03 (1.77-2.33)	2.50 (2.00-3.13)
**Gastric cancer (151) [3]**	1.30 (1.23-1.37)	1.53 (1.44-1.2)
**Pancreatic cancer (157) [4]**	1.20 (1.10-1.20)	1.80 (1.70-1.90)
**Laryngeal cancer (161) [2]**	4.65 (3.35-6.45)	6.98 (3.14-15.5)
**Lung cancer (162) [2]**	3.85 (2.77-5.34)	8.96 (6.73-12.10)
**Bladder cancer (188) [2]**	1.72 (1.46-2.04)	2.77 (2.17-3.54)
**IHD (410-414) [5]**	Age < 65: 1.45 (1.41-1.50)	Age < 65: 3.06 (3.00-3.13)
	Age ≥ 65: 1.12 (1.07-1.16)	Age ≥ 65: 1.66 (1.59-1.74)
**Stroke (430-438) [5]**	Age < 65: 1.30 (1.12-1.50)	Age < 65: 3.12 (2.80-3.47)
	Age ≥ 65: 1.15 (1.07-1.24)	Age ≥ 65: 1.65 (1.52-1.79)
**Arteriosclerosis (440-448) [5]**	1.82 (1.70-1.95)	2.54 (2.42-2.67)
**Pneumonia and influenza (480-487) [5]**	1.29 (1.15-1.45)	1.47 (1.33-1.61)
**COPD (490-492, 496) [5]**	6.70 (6.20-7.20)	9.80 (9.20-10.20)

COPD: chronic obstructive pulmonary disease, IHD: ischaemic heart disease.

Supplementary Table 3 – Sensitivity analysis of hospitalisations and expenditures attributable to smoking, stratified by disease, using relative risks from Carter et al. [6] (Italy, 2018)

Disease	PAF hospitalisations	Attributable hospitalisations	PAF expenditure	Attributable expenditure
**Oropharyngeal cancer**	59.62%	5,808	59,76%	€ 27,674,642
**Esophageal cancer**	56.38%	2,540	56.67%	€ 12,855,218
**Gastric cancer**	23.66%	4,701	23.59%	€ 28,054,514
**Pancreatic cancer**	15.66%	3,837	15.80%	€ 19,436,858
**Laryngeal cancer**	83.75%	6,494	83.63%	€ 35,266,729
**Lung cancer**	87.72%	59,898	87.80%	€ 272,572,151
**Bladder cancer**	54.90%	42,797	54.95%	€ 138,985,439
**IHD**	37.12%	97,712	37.71%	€ 582,489,872
**Stroke**	19.63%	49,258	20.38%	€ 274,671,833
**Arteriosclerosis**	44.77%	38,565	46.57%	€ 260,908,848
**Pneumonia and influenza**	23.37%	31,738	23.37%	€ 114,167,591
**COPD**	85.24%	30,246	85.89%	€ 75,275,728
**Total**	**37.96%**	**373,621**	**37.31 %**	**€ 1,842,359,424**

Attributable expenditure: expenditure (€) attributable to tobacco smoking, Attributable hospitalisations: number of hospitalisations attributable to tobacco smoking, COPD: chronic obstructive pulmonary disease, IHD: ischaemic heart disease, PAF: Population Attributable Fraction

Supplementary Figure 1 - Hospitalisation rates of 12 tobacco-related diseases, per million inhabitants (Italy, 2018)

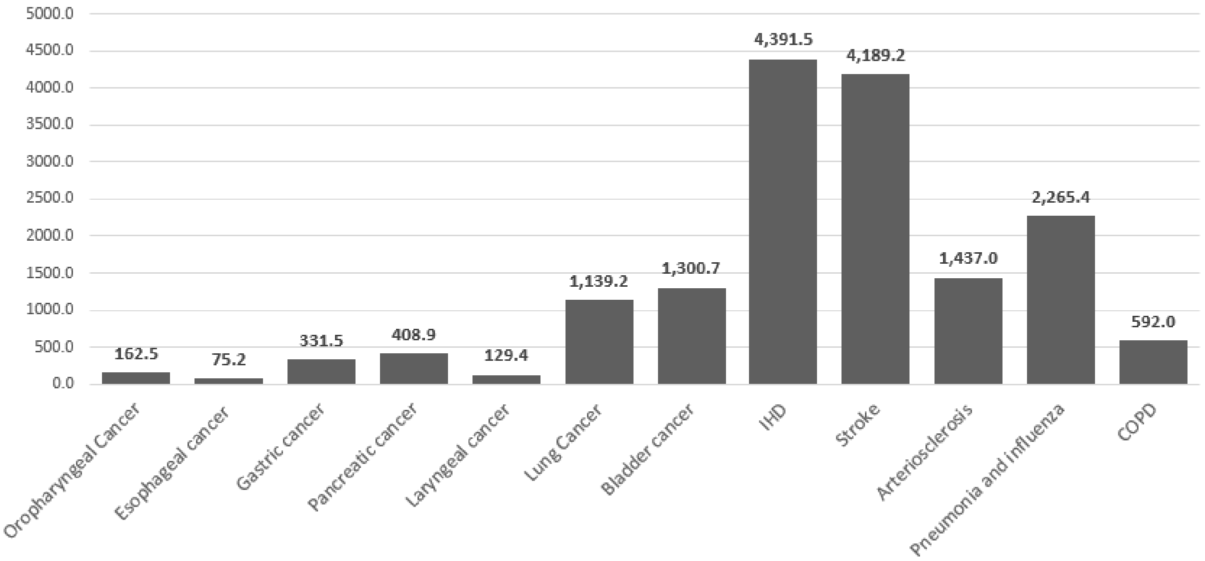

COPD: chronic obstructive pulmonary disease, IHD: ischaemic heart disease.

References

1. ISTAT. I.Stat - your direct access to the Italian Statistics. Available at http://dati.istat.it/?lang=en. Accessed December 14, 2023.

2. Gandini S, Botteri E, Iodice S, Boniol M, Lowenfels AB, Maisonneuve P, et al. Tobacco smoking and cancer: a meta-analysis. *International journal of cancer.* 2008;122(1):155-164. doi: 10.1002/ijc.23033

3. Rota M, Possenti I, Valsassina V, Santucci C, Bagnardi V, Corrao G, et al. Dose-response association between cigarette smoking and gastric cancer risk: a systematic review and meta-analysis. *Gastric Cancer.* 2024. doi: 10.1007/s10120-023-01459-1

4. Lugo A, Peveri G, Bosetti C, Bagnardi V, Crippa A, Orsini N, et al. Strong excess risk of pancreatic cancer for low frequency and duration of cigarette smoking: A comprehensive review and meta-analysis. *Eur J Cancer.* 2018;104:117-126. doi: 10.1016/j.ejca.2018.09.007

5. English D. R., Holman C. D. J., Milne E., Winter M. G., Hulse G., Codde J. P., et al. The quantification of drug caused morbidity and mortality in Australia. *Australian Government Publishing Service.* 1995;2.

6. Carter BD, Abnet CC, Feskanich D, Freedman ND, Hartge P, Lewis CE, et al. Smoking and mortality--beyond established causes. *N Engl J Med.* 2015;372(7):631-640. doi: 10.1056/NEJMsa1407211

©2024 Possenti I. et al.
